# Comparative evaluation of open flap debridement with or without injectable platelet-rich fibrin in chronic periodontitis treatment

**DOI:** 10.6026/973206300220436

**Published:** 2026-01-31

**Authors:** Sakshi Verma, Vandana Saranga, Supreet Kaur, Sahib Tej Singh, Vidhuta Sareen, Simranjit Kaur, Simrita Singh

**Affiliations:** 1 Department of Periodontology, Sri Guru Ram Das Institute of Dental Sciences and Research, Amritsar, Punjab, India

**Keywords:** Periodontitis, platelet-rich fibrin, open flap debridement, intrabony defects, regenerative therapy

## Abstract

Periodontitis, particularly generalized Stage III Grade B periodontitis with intrabony defects, presents significant challenges in treatment, requiring innovative adjunctive therapies to enhance the effectiveness of conventional treatments such as open flap debridement (OFD). Hence, twenty-four patients were randomized to receive OFD alone or OFD + i-PRF. The results showed significant improvements in clinical parameters and radiographic defect fill at 6 months in i-PRF group. I-PRF demonstrated superior outcomes in probing pocket depth reduction and relative attachment level gain. It offers a promising, biocompatible, cost-effective approach for periodontal regeneration. Thus, the of effect i-PRF's as an adjunct to open flap debridement, showing significant improvements in clinical and radiographic outcomes for Stage III Grade B periodontitis with intrabony defects is reported.

## Background:

Periodontitis is a chronic inflammatory disease affecting the supporting structures of the teeth, particularly the periodontal ligament and alveolar bone, caused by specific pathogens like *Porphyromonas gingivalis*, *Tannerella forsythia* and *Treponema denticola*. These bacteria trigger an inflammatory cascade that results in the release of inflammatory mediators, including prostaglandins (PGs), cytokines and matrix metalloproteinases (MMPs), which contribute to tissue degradation and bone resorption [1]. Periodontitis manifests as gingival inflammation, increased probing depth and eventual loss of connective tissue and bone around affected teeth. Genetic factors, such as chromosomal abnormalities and environmental factors that weaken immune responses, contribute to the variability in disease presentation and progression [2]. Regeneration in the context of periodontal therapy refers to the restoration of lost or damaged tissues, aiming to rebuild the architecture and function of the periodontium [3]. Bone grafting, a cornerstone in regenerative therapy, plays a crucial role by providing a scaffold for new bone formation and enhancing healing in periodontal defects. Various biomaterials based on endogenous regenerative technology (ERT) have been used for periodontal regeneration, including autografts, allografts, xenografts and synthetic materials [4]. Open-flap debridement (OFD) is one of the earliest surgical procedures used for periodontal therapy, demonstrating favorable outcomes for treating intrabony defects. However, OFD alone has limited potential for regenerating lost periodontal structures [5]. Studies have shown that combining OFD with bone grafts significantly improves regenerative outcomes. Bone graft materials can be autologous (from the patient), allogenic (from human donors), xenogenic (from animals), or synthetic [6]. While autografts provide osteogenic potential, other graft types primarily serve as scaffolds, supporting bone growth. However, challenges such as donor site morbidity, immune reactions and slow resorption of these grafts have prompted the search for biologically enhanced alternatives [7]. Recent advancements in regenerative medicine have shifted the focus towards biomaterials that not only provide structural support but also enhance healing at the molecular level. Autologous platelet concentrates, such as Platelet-Rich Fibrin (PRF), have gained attention for their ability to release growth factors and cytokines directly at the site of injury, leveraging the body's natural healing mechanisms [8]. PRF was initially used in oral and maxillofacial surgery and has since expanded to other medical fields due to its biocompatibility and effectiveness in promoting tissue healing [9]. Unlike Platelet-Rich Plasma (PRP), PRF is an autologous, anticoagulant-free product, making it more biocompatible and reducing the risk of immune reactions [10]. In 2017, a modified form of PRF, injectable PRF (i-PRF), was introduced. This variant offers higher concentrations of growth factors such as insulin-like growth factor (IGF-1), epidermal growth factor (EGF) and platelet-derived growth factor (PDGF-AA/AB) [11]. I-PRF has been shown to enhance fibroblast migration and support tissue regeneration through a sustained release of growth factors over 10 days. Its liquid fibrin matrix not only contains a high concentration of platelets but also promotes the migration of leukocytes and fibroblasts, further facilitating tissue repair [12]. Moreover, i-PRF's biodegradability and lack of long-term complications make it a promising material for minimally invasive regenerative therapies. Given its regenerative capacity and clinical benefits, i-PRF has been proposed as an adjunct to conventional OFD to address the limitations of traditional methods [13]. Therefore, it is of interest to determine the therapeutic efficacy of i-PRF in the management of intrabony defects in patients with chronic periodontitis, advancing minimally invasive, biologically driven approaches in periodontal regeneration.

## Methodology:

This study included twenty-four systemically healthy patients, comprising 17 males and 7 females, who were randomly assigned to two groups in a 6-month follow-up clinical trial conducted at the Department of Periodontology and Oral Implantology, Sri Guru Ram Das Institute of Dental Sciences and Research, Amritsar. The research protocol was approved by the Institutional Ethical Committee and informed consent was obtained from all participants. Inclusion criteria consisted of patients with generalized Stage III Grade B periodontitis, who presented with intrabony defects showing a probing depth (PD) and clinical attachment level (CAL) of ≥5mm, along with radiographic evidence of intrabony defects ≥3mm. Patients with systemic diseases, medications affecting periodontal therapy, tobacco use, previous periodontal therapy in the last 6 months, or those who were pregnant or lactating were excluded from the study. At the first visit, all patients underwent full-mouth scaling and root planing (SRP) as part of Phase 1 therapy. Proper oral hygiene instructions were also given. After six weeks, periodontal evaluation confirmed the sites suitable for study inclusion. The selected sites were then randomly assigned to either the control group (OFD alone) or the test group (OFD + i-PRF). The clinical parameters=plaque index (PI), gingival index (GI), probing pocket depth (PPD), relative attachment level (RAL) and intrabony defect fill (IBD)-were recorded at baseline, three months and six months. A custom-made acrylic stent and a Williams's periodontal probe were used for standardized measurements and radiographic assessment of bone defect fill was performed using a calibrated grid. I-PRF was prepared by drawing 10 mL of venous blood from each patient and centrifuging it at 700 rpm for 3 minutes, resulting in a distinct plasma layer containing platelet-rich fibrin. This fibrin concentrate, rich in bioactive components, was then immediately applied to the targeted defect sites. In the surgical procedure, patients rinsed with 0.12% chlorhexidine digluconate before local anesthesia was administered. Crevicular incisions were made and a full-thickness mucoperiosteal flap was reflected to expose the osseous defect. After thorough debridement using Gracey curettes, i-PRF was applied in the test group, while the control group received OFD alone. Suturing was done to reposition the flap and the surgical site was covered with a non-eugenol periodontal dressing. Post-operatively, patients were prescribed amoxicillin and ibuprofen, along with chlorhexidine mouthwash. Patients were advised to avoid mechanical plaque control at the surgical site for 7-10 days and to follow a soft diet for the first 48 hours. Clinical and radiographic evaluations were conducted at three and six months to assess changes in periodontal parameters and bone fill. The statistical analysis was performed using SPSS version 24. Data were analyzed using t-tests to compare within groups and unpaired t-tests to compare the control and test groups at baseline, three months and six months. Chi-square tests were also used to analyze the association between categorical variables.

## Results:

Wound healing was uneventful for all treated cases. Soft tissues heal within normal limits and no significant visual differences were noted between the treatment groups. A statistically significant reduction in PI and GI was observed in both test and control group at 6 months post-operatively. However, the difference between the test and control sites was statistically significant. Intragroup and intergroup comparisons showed statistically significant reduction with PPD and RAL and statistically significant bone fill was also observed in intrabony defect fill (IBD) when intragroup and intergroup comparisons were made.

**Figure 1:**
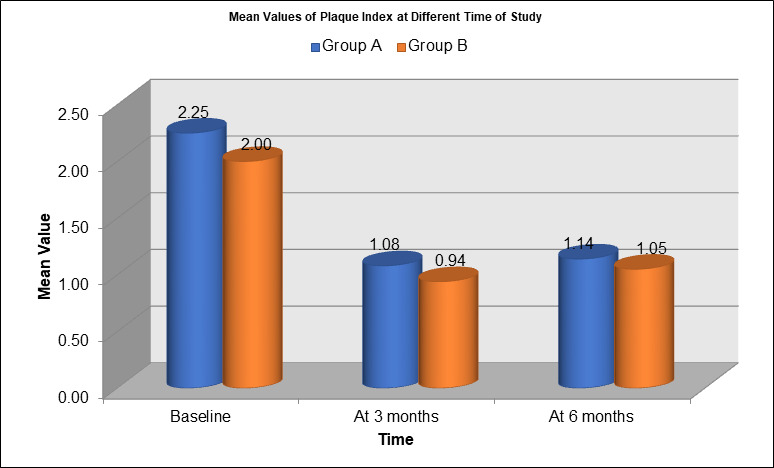
Mean values of the Plaque Index (PI)

**Figure 2:**
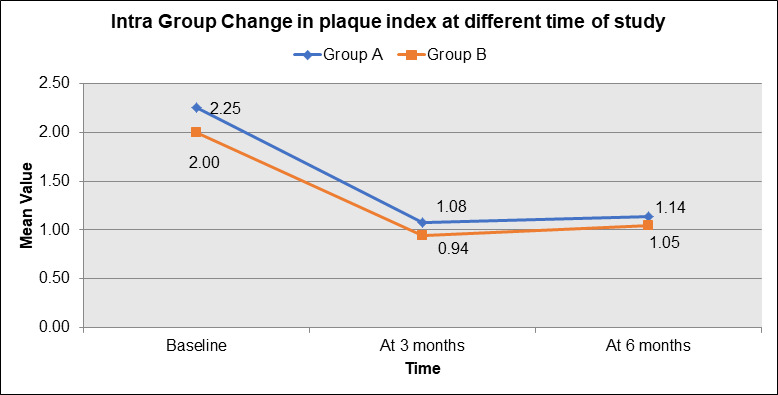
Intra-group change in plaque index at different time of study

**Figure 3:**
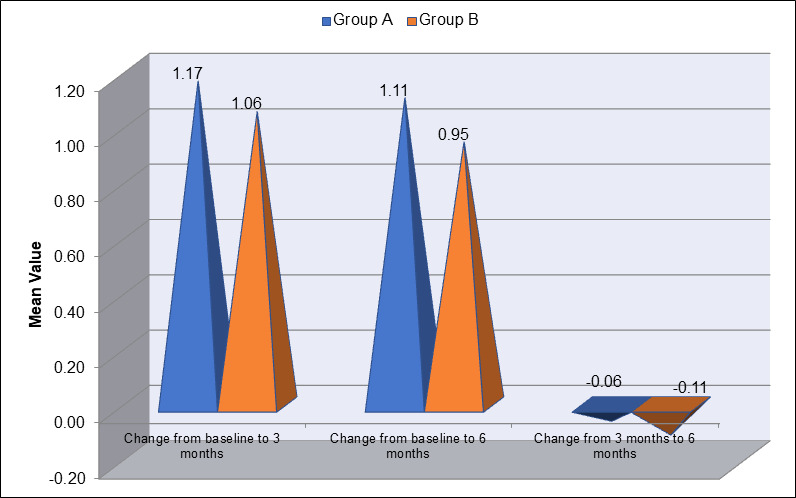
Inter-Group Comparison of Change in plaque index

**Figure 4:**
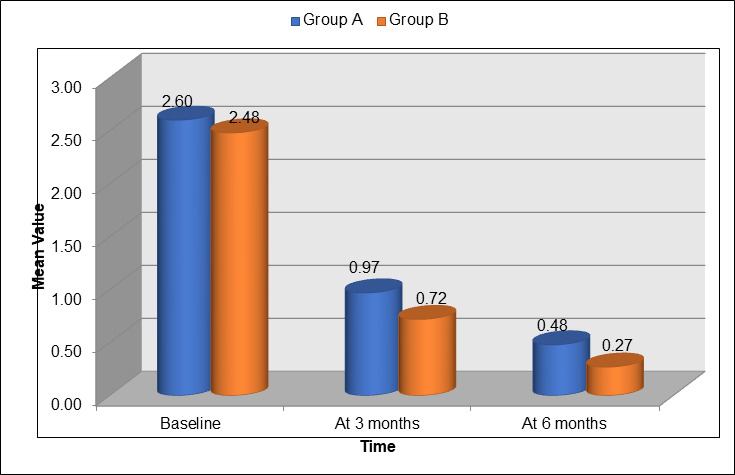
Gingival index at different times of the study

Figure 1 depicts the mean values of the Plaque Index (PI) at different time points for two groups, Group A (blue) and Group B (orange). At baseline, Group A had a higher mean PI of 2.25, while Group B had a slightly lower mean of 2.00. After 3 months, both groups showed improvement in plaque control, with Group A's mean PI reducing to 1.08 and Group B showing a more significant reduction to 0.94. By 6 months, both groups continued to demonstrate further improvement, with Group A's mean PI reaching 1.14 and Group B's PI improving slightly further to 1.05. Overall, the data shows a consistent decrease in plaque index for both groups, with Group B demonstrating better plaque control across all time points, especially at 3 and 6 months. Figure 2 illustrates the intra-group changes in Plaque Index (PI) for both Group A (blue) and Group B (orange) at different time points of the study. At baseline, Group A had a higher mean PI of 2.25 compared to Group B's 2.00. Both groups showed improvement by 3 months, with Group A's PI dropping to 1.08 and Group B's reducing further to 0.94. By 6 months, Group A's PI increased slightly to 1.14, while Group B's PI remained relatively stable at 1.05. The graph demonstrates that both groups had a significant reduction in PI over time, but Group B showed a more consistent and greater improvement in plaque control compared to Group A, particularly by the 3-month and 6-month marks. Figure 3 illustrates the inter-group comparison of the change in Plaque Index (PI) between Group A (blue) and Group B (orange) at three different time intervals: baseline to 3 months, baseline to 6 months and 3 months to 6 months. From baseline to 3 months, Group A showed a larger reduction in PI (mean change of 1.17) compared to Group B, which had a mean change of 1.06. However, by 6 months, the reduction in PI for Group A was slightly lower (mean change of 1.11), while Group B showed a smaller change of 0.95 from baseline to 6 months. When comparing the changes between 3 and 6 months, both groups showed minimal reductions, with Group A having a change of -0.06 and Group B a slightly greater change of -0.11. This data indicates that although Group A showed more significant initial improvement, Group B exhibited a more consistent reduction in plaque index over time, especially between 3 and 6 months. Figure 4 displays the mean values of the Gingival Index (GI) for Group A (blue) and Group B (orange) at different time points during the study. At baseline, Group A had a mean GI of 2.60, while Group B had a slightly lower mean value of 2.48. At 3 months, both groups showed improvement, with Group A's GI reducing to 0.97 and Group B's GI decreasing to 0.72. By 6 months, Group A's GI further decreased to 0.48, while Group B showed a greater improvement, with a mean GI of 0.27. This data demonstrates that both groups showed significant improvements in gingival health, but Group B exhibited a more consistent reduction in gingival inflammation across all time points, particularly by 6 months.

**Figure 5:**
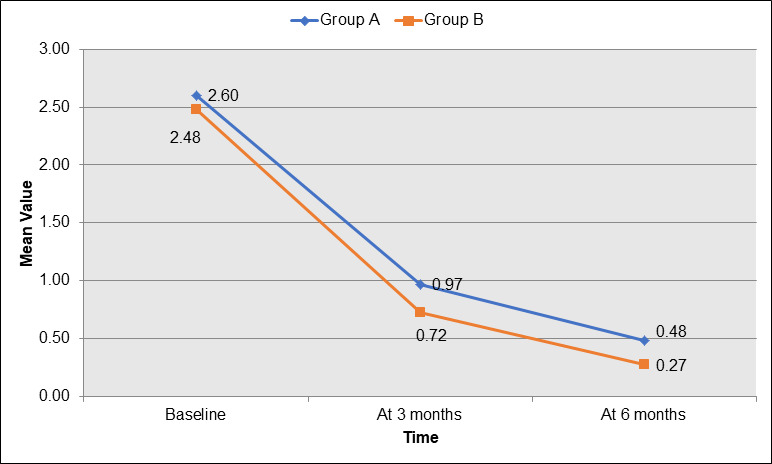
Intra-group change in gingival index at different time of study

Figure 5 illustrates the intra-group changes in the Gingival Index (GI) over time for both Group A (blue) and Group B (orange). At baseline, Group A had a mean GI of 2.60, while Group B had a slightly lower mean of 2.48. Over the study period, both groups showed significant improvement in gingival health. At 3 months, Group A's GI decreased to 0.97 and Group B's GI improved to 0.72. By 6 months, Group A's GI further reduced to 0.48, while Group B showed the greatest improvement with a mean GI of 0.27. This data highlights that both groups experienced a significant reduction in gingival inflammation, with Group B showing a more consistent and greater reduction in GI at all-time points, particularly by 6 months.

**Figure 6:**
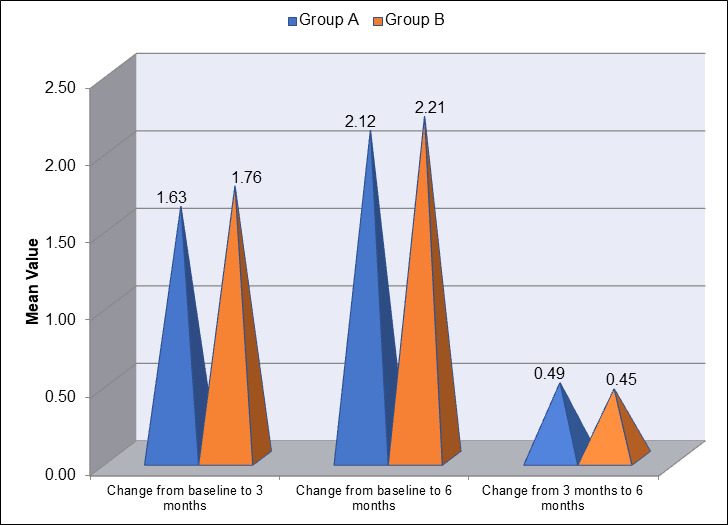
Inter-group comparison of change in gingival index

Figure 6 presents the inter-group comparison of the change in Gingival Index (GI) between Group A (blue) and Group B (orange) at different time intervals: baseline to 3 months, baseline to 6 months and 3 months to 6 months. From baseline to 3 months, Group A showed a mean change of 1.63, while Group B demonstrated a slightly larger change of 1.76. Between baseline and 6 months, both groups showed further improvement, with Group A's mean change being 2.12 and Group B's change being slightly greater at 2.21. When comparing the change from 3 months to 6 months, Group A had a mean change of 0.49, while Group B showed a smaller change of 0.45. This data highlights that Group B experienced a greater reduction in GI overall, especially in the early stages of the study, indicating a more significant improvement in gingival health over time compared to Group A. The data in Table 1 shows the Probing Pocket Depth (PPD) changes for both Group A and Group B at different time points (baseline, 3 months and 6 months). At baseline, Group A had a mean PPD of 6.90, while Group B had a slightly higher mean of 7.11. Both groups showed improvement by 3 months, with Group A's PPD decreasing to 5.97 and Group B's decreasing to 3.86. By 6 months, Group A's PPD was 4.35 and Group B's was 3.56, indicating continued improvement. In terms of intra-group changes, Group A saw a reduction from 6.90 at baseline to 4.35 at 6 months, with the largest improvement happening at 3 months. Group B showed a more significant reduction from 7.11 at baseline to 3.56 at 6 months, with a notable decrease at 3 months as well. The inter-group comparison revealed that from baseline to 3 months, Group A had a reduction of 3.25, while Group B experienced a slightly greater reduction of 3.55. From baseline to 6 months, Group A showed a change of 2.55, while Group B had a reduction of 3.55. The change from 3 months to 6 months was smaller for both groups, with Group A showing a change of 0.93 and Group B only 0.32. Overall, both groups showed significant improvements in probing pocket depth, with Group B consistently demonstrating a more substantial reduction, particularly in the initial stages. Table 2 presents the Relative Attachment Level (RAL) and Intrabony Defect Fill for both Group A (blue) and Group B (orange) at different time points during the study. At baseline, Group A had a mean RAL of 9.90, while Group B had a slightly higher mean of 10.11. Both groups showed improvement over time: by 3 months, Group A's mean RAL reduced to 8.87 and Group B's dropped to 6.86. By 6 months, Group A's RAL improved to 7.35, while Group B's reached 6.56. In terms of Intrabony Defect Fill, at baseline, Group A had a mean defect fill of 5.21, while Group B had 5.00. Both groups demonstrated improvement by 3 months, with Group A's defect fill increasing to 4.02 and Group B's to 3.57. By 6 months, Group A's defect fill reached 3.37, while Group B's was 2.57. When comparing the changes in RAL, Group B consistently showed larger improvements, particularly from baseline to 6 months, while Group A had a more gradual reduction in changes between 3 and 6 months. Regarding defect fill, both groups showed significant improvements, but Group B had a greater increase in defect fill by 6 months. This data highlights the effective improvement in periodontal health for both groups, with Group B generally showing better outcomes in both RAL and intrabony defect fill across the study period (Table 3).

## Discussion:

The present study was aimed at evaluating the clinical efficacy and regenerative potential of autograft injectable platelet-rich fibrin (i-PRF) in the management of intrabony periodontal defects in patients with Generalized Stage III Grade B periodontitis. In assessing the success of these treatment methods, complete closure of the defect is desirable. Therapeutic results can be measured by PPD and RAL, bone regeneration and evidence of histologic periodontal regeneration and evidence. The ideal goal for periodontal therapy is the reconstitution of bone and connective tissue attachment that has been destroyed by the disease process. The uneventful healing in the patients is in agreement with studies by various authors thus supporting the excellent properties of autologous PRF to enhance periodontal wound healing [14]. Although changes in periodontal tissues are significant, tooth loss primarily results from alveolar bone destruction. Bone undergoes constant remodeling through resorption and formation, processes typically in balance under healthy conditions. Disruption of this equilibrium is closely linked to bone loss. In periodontitis, bone destruction often follows the apical spread of gingival inflammation into supporting structures. While all periodontitis is preceded by gingivitis, not all gingivitis cases progress to periodontitis [15]. Microbial dysbiosis is central to disease progression. The "red complex"-*Porphyromonas gingivalis*, *Tannerella forsythia* and *Treponema denticola*-is strongly associated with alveolar bone destruction [16]. Aggregatibacter actinomycetemcomitans, linked to aggressive periodontitis, exacerbates bone loss through leukotoxin production. These pathogens activate host immune responses, notably RANKL-mediated osteoclastogenesis, resulting in bone resorption [17, 18]. The pattern of bone loss depends on microbial virulence, host response and local anatomical factors, presenting as either horizontal or vertical bone loss. Horizontal bone loss reflects uniform resorption along the alveolar crest, whereas vertical defects result from localized inflammatory destruction [18]. There was a mean reduction in plaque index and gingival index score in test and control group from baseline to 6 months post-surgery both within and between groups which was statistically not significant. This implied that the participants of the study exercised good oral home care. The mean probing depth reduction in Group A (OFD) and Group B (OFD + i-PRF) were 6.90 ± 0.322 mm and 7.11 ± 1.006 mm respectively which reduced to 5.97 ± 0.484 and 3.86 ± 0.427 mm respectively at the end of 3 months and 4.35 ± 0.454 and 3.56 ± 0.451 at the end of 6 months post-treatment upon intragroup comparison. In the present study the mean value of relative attachment level in Group A (OFD) and Group B (OFD + i-PRF) was 9.90 ± 0.322 mm and 10.11 ± 1.056 mm respectively which gained to 8.97 ± 0.484 mm and 6.86 ± 0.427 mm respectively at the end of 3 months and 7.35 ± 0.454 mm and 6.56 ± 0.451 mm respectively at the end of 6 months post-treatment. Another study reported a CAL gain of 3.69 ± 0.44 mm on test sites compared to 2.13 ± 0.43 mm on control sites at end of 9 months. The gain in CAL appears to be similar to studies that reported mean improvement in CAL 3.69 ± 0.44 and 3.31 ± 1.76 mm in 9 months. The mean intrabony defect fill upon intragroup comparison, at baseline, the mean value of IBD were 5.21 ± 0.619 mm (Group A) and 5.00 ± 0.656 mm (Group B), which showed a gain of 4.02 ± 0.756 mm in Group A and 3.57 ± 0.760 mm in Group B by 3 months which was statistically non- significant (p>0.05). However, by 6 months, it showed further gain of 3.87 ± 0.739 mm in Group A and 2.47 ± 0.460 mm in Group B, which was statistically significant (p<0.001). Other authors reported a mean percentage fill at of 46.92% 12 and 48.26%± 5.72% at end of 9 months. However, they reported mean percentage fill in control sites (OFD) 1.80% and 1.56% at 9 months, which is much lower than seen in the present study, where mean percentage fill of 16.41 ±7.3821 [19]. Likewise, in an interesting study, 90 intrabony defects were randomized into 3 groups and were treated with PRF, PRP and OFD alone. The PD reduction of 3.77 ±1.19mm and 3.77 ±1.07mm was observed in the PRF and PRP groups in comparison to 2.97±0.93mm in OFD group. A CAL gain of 3.17 ±1.29mm and 2.93 ±1.08mm was observed in the PRF and PRP groups in contrast to 2.83±0.91mm in the OFD group. The bone fill percent was 54.41%±11.39, 56.85%±14.01 and 1.56%±15.12 in PRF, PRP and OFD groups, respectively [20].

## Conclusion:

We show that while open flap debridement (OFD) alone is effective in treating intrabony defects, the adjunctive use of injectable platelet-rich fibrin (i-PRF) significantly enhances clinical and radiographic outcomes. I-PRF promoted greater bone fill and probing depth reduction, suggesting superior regenerative potential. Its autologous origin, safety, ease of use and cost-effectiveness make it a valuable adjunct to conventional periodontal therapy. Future studies with larger sample sizes and extended follow-up are warranted to validate these findings and assess long-term benefits.

## Figures and Tables

**Table 1:** Probing Pocket Depth (PPD) data for Group A and Group B at different time points

**Group**	**Baseline Mean PPD**	**3 Months Mean PPD**	**6 Months Mean PPD**	**Change from Baseline to 3 Months**	**Change from Baseline to 6 Months**	**Change from 3 Months to 6 Months**
Group A	6.9	5.97	4.35	3.25	2.55	0.93
Group B	7.11	3.86	3.56	3.55	3.55	0.32

**Table 2:** Relative Attachment Level (RAL) and Intrabony Defect Fill for both Group A and Group B

**Group**	**Baseline Mean RAL**	**3 Months Mean RAL**	**6 Months Mean RAL**	**Change from Baseline to 3 Months**	**Change from Baseline to 6 Months**	**Change from 3 Months to 6 Months**
Group A	9.9	8.87	7.35	3.08	2.55	1.62
Group B	10.11	6.86	6.56	3.38	3.55	0.3

**Table 3:** Intrabony defect fill

**Group**	**Baseline Mean IBD**	**3 Months Mean IBD**	**6 Months Mean IBD**	**Change from Baseline to 3 Months**	**Change from Baseline to 6 Months**	**Change from 3 Months to 6 Months**
Group A	5.21	4.02	3.37	1.19	1.84	0.65
Group B	5	3.57	2.57	1.42	2.43	1
